# Wax on, wax off: a case report discussing a potential pitfall of dissolvable bone wax substitutes such as Ostene® in neurosurgery

**DOI:** 10.1007/s00701-025-06473-z

**Published:** 2025-03-08

**Authors:** Madeleine Louise Buck, Kristian John Bulluss, Paul Daniel Smith

**Affiliations:** 1 Department of Neurosurgery, St Vincent’s Hospital Melbourne, Fitzroy, VIC Australia; 2 Department of Surgery, University of Melbourne, Parkville, VIC Australia

**Keywords:** Bone wax, Ostene, Resorbable, Complication, Skull base repair, CSF leak

## Abstract

Bone wax alternatives, such as Ostene®, are marketed as substitutes for traditional bone wax, offering the benefits of resorbability, improved bone osteogenesis, and reduced inflammatory reactions, granulomas, and infection risks. However, the limitations of these agents in cranial neurosurgery, particularly for sealing exposed air cells during skull base repair, are underreported. We present an early post-operative case of subcutaneous emphysema, pneumocephalus, and bone flap infection following the failure of Ostene® to provide a durable seal of air cells after temporal craniotomy and tumour resection. This highlights a limitation of Ostene® and other water-soluble bone haemostasis agents in cranial neurosurgery.

## Introduction

Bone wax, predominantly composed of beeswax with softening additives, has been used as a haemostatic agent in cranial neurosurgery for centuries. Its use was popularised by Dr. Victor Horsley, who described its formula in 1892. However, its application in neurosurgery was first described in 1864, when Dr. Henri Ferdinand Dolbeau used it during the extirpation of a frontal sinus osteoma in Paris [2, 4]. Bone wax acts as a mechanical tamponade, coating bone surfaces to provide effective haemostasis [2]. It is widely used in neurosurgery, orthopaedics, and cardiothoracic surgery due to its affordability and efficacy. However, it has been criticised for inducing inflammation, granuloma formation, impaired bone healing, and increased risks of infection [5, 8–11]. These issues are attributed to its insoluble nature, which leaves a permanent foreign body in situ, leading to inflammatory reactions and reduced bacterial clearance from bone [2]. To mitigate these complications, alternatives such as Ostene® (Baxter International Inc., Deerfield, IL, USA) —a water-soluble bone haemostasis agent made of alkylene oxide copolymers—have been developed.

Unlike traditional wax, these alternatives are resorbable, dissolving usually within 24–48 h of application [8, 10], and provide a temporary mechanical barrier for intraoperative haemostasis, potentially reducing the long-term complications associated with permanent foreign material [5, 9]. While these properties may be advantageous in many surgical contexts, their use in neurosurgery raises concerns, particularly for sealing air cells during skull base repair to prevent cerebrospinal fluid (CSF) leakage, pneumocephalus, and intracranial infections. We present a case illustrating the use of Ostene® in this context, where its dissolution resulted in pneumocephalus, CSF leak, and bone flap infection, necessitating revision surgery and a prolonged course of antibiotics.

## Case report

**Fig. 1:**
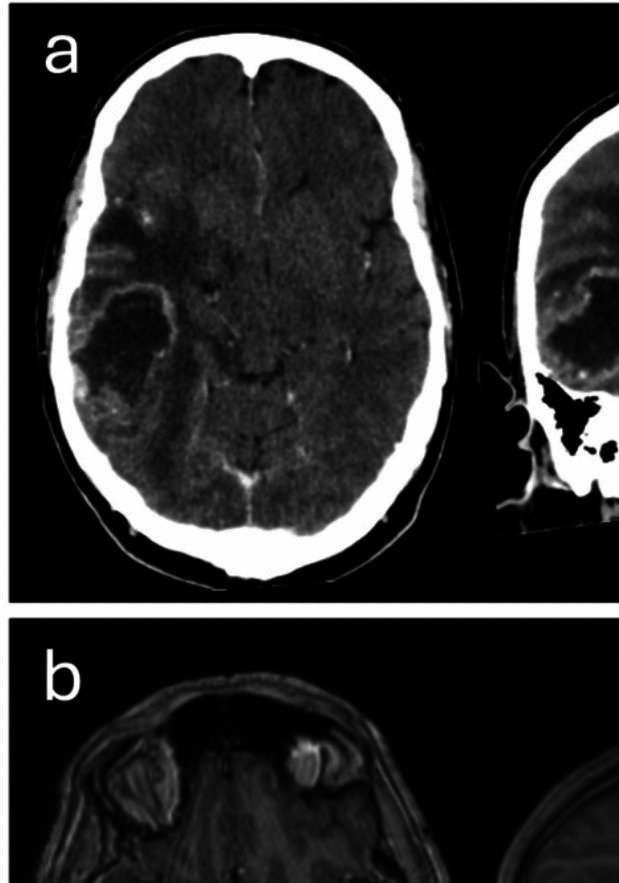
Axial and coronal (**a**) CT and (**b**) MRI sections from imaging performed preoperatively, on the day of presentation to hospital, revealing a contrast enhancing lesion of the right temporal lobe

A 63-year-old male presented with worsening headaches, ataxia, and cognitive dysfunction. His medical history included hypertension and a transient ischemic attack in 2020, for which he was taking aspirin and an antihypertensive. Computed tomography (CT) and magnetic resonance imaging (MRI) of the brain, performed on presentation, revealed a multifocal contrast-enhancing lesion in the right posterior temporal lobe with an additional enhancing focus at the temporal pole, consistent with a high-grade glioma (Fig. 1a and b).

**Fig. 2:**
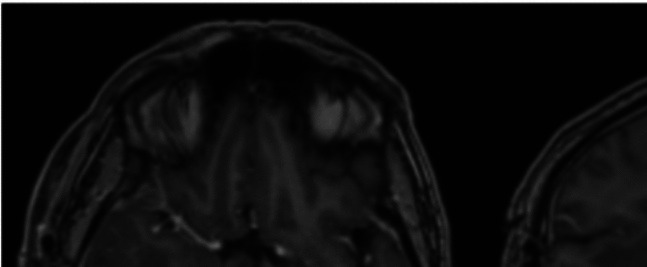
Postoperative day one axial and coronal MRI sections revealing satisfactory postoperative appearances and minimal wound swelling

The patient was admitted to hospital and underwent a craniotomy and tumour debulking three days later. Temporal bone air cells were exposed intraoperatively and sealed with Ostene®. Histopathological examination confirmed a diagnosis of World Health Organisation (WHO) Grade 4 Glioblastoma, IDH wildtype. Postoperative recovery was unremarkable, with no neurological deficits, and imaging performed on post operative day one showed no complications (Fig. 2).

The patient was discharged on postoperative day three with a healthy wound and no neurological deficits.

**Fig. 3:**
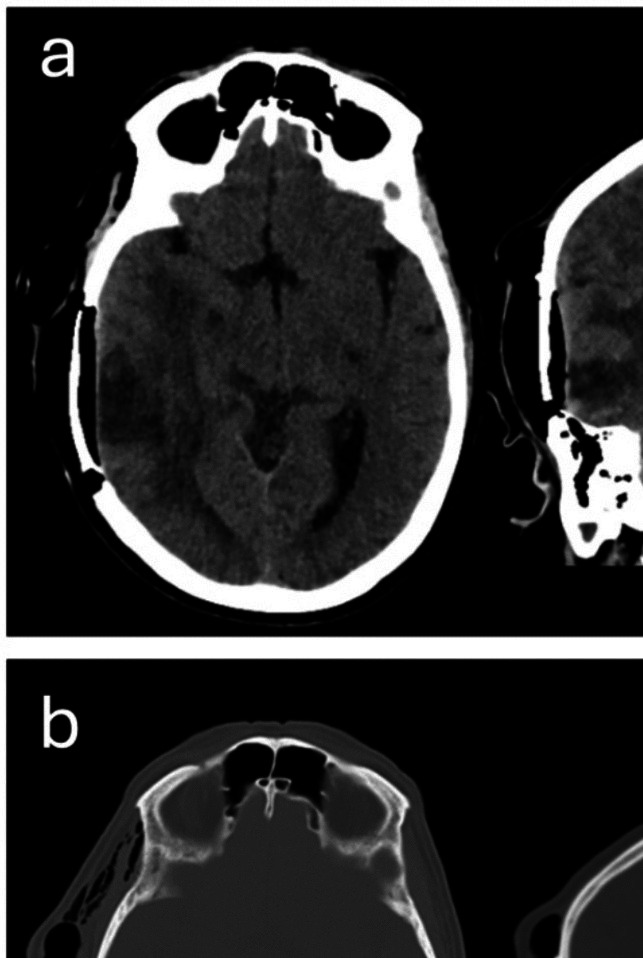
Axial and coronal CT sections with both (**a**) brain and (**b**) bone windowing, performed at time of re-presentation on postoperative day eight and prior to second surgery, demonstrating extensive subcutaneous emphysema, pneumocephalus and fluid in the right-sided mastoid air cells

On postoperative day eight, the patient returned with swelling around the craniotomy site, a sensation of pressure in that region and crackling in his right ear. He was afebrile and exhibited no otorrhoea, rhinorrhoea, or headache. Examination revealed a healthy wound with underlying fluctuance without leak or dehiscence. CT imaging on that day demonstrated subcutaneous emphysema, pneumocephalus, and a right-sided mastoid effusion (Fig. 3).

The presumptive diagnosis was of exposed mastoid air cells, and the decision was made to proceed to surgical repair via redo craniotomy the following day. At surgery, exposed mastoid air cells were noted, and there was no evidence of the Ostene® product. The air cells were waxed extensively with conventional bone wax. A layered repair followed with TachoSil® and Tisseel® reinforcement. There was no evidence of extracranial or intracranial infection, although swabs were taken and sent to microbiology for analysis. The bone flap was, therefore, replaced, followed by routine closure.

The patient recovered well postoperatively without neurological deficit; however, the intraoperative swabs grew Staphylococcus aureus and Staphylococcus epidermidis, and the patient has been commenced on a prolonged course of intravenous and now oral antibiotic therapy. The decision was made to leave the infected bone flap in situ and treat with prolonged antibiotic therapy to avoid a further surgery for bone flap removal and potential delay to adjuvant therapy. He remained afebrile with low inflammatory markers throughout his admission and was discharged on postoperative day five. The patient subsequently commenced adjuvant therapy with radiation and concomitant temozolomide without delay in line with the Stupp regimen standard of care.

## Discussion

Our case is, to our knowledge, the first published case highlighting a potential limitation of Ostene® and perhaps other similar water-soluble bone haemostasis agents when used in neurosurgical applications beyond haemostasis, such as to control potential CSF leaks. There have been many attempts over the last century to produce a bone wax substitute, which maintains the advantages of the wax but minimises its complications. However, none have succeeded in the long term [2, 3, 11]. Bone wax is advantageous as it is malleable, easy to handle, moulds to irregular bony surfaces, is cheap, and has a long shelf life [1, 2]. The inert nature of wax ensures it provides a durable seal on bone and adheres strongly, and it is backed by a long history of effective clinical use [2, 7, 11]. However, bone wax is not without disadvantages and complications including foreign body reaction and granuloma growth, impaired osteogenesis, promotion of infection and thrombosis induction have been described arising from its use [5, 8–11]. It is contended that these complications have arisen, mainly due to the poor biocompatibility of bone wax and its inert nature, and so development of a substitute has focused on absorbability.

Ostene® synthetic putty has been developed and is marketed as an alternative to traditional bone wax. It has been compared with bone wax in preclinical studies [5, 8–10]. Ostene® is not a wax itself. It is a synthetic putty comprised of alkylene oxide copolymers. However, it is advertised as having the same ability to act as a mechanical haemostat on bony surfaces which is temporary and dissolves over 24- 48 h [5, 9], reducing complications associated with traditional wax. While these advantages are often highlighted, they might not be born out in clinical practice, as most supporting data comes from preclinical studies or animal models [5, 8–10].^.^ Our case illustrates another practical problem arising from its absorbable nature: its insufficient durability as a sealing agent thus leading to the development of pneumocephalus after its use for mastoid air cell sealing.

During skull base repair and reconstruction, durability of the sealing wax is critical in maintaining a long-term barrier to prevent CSF leak or infection from exposed air cells and air sinuses. This technique is well described and widely utilised in neurosurgery [2, 3, 7]. The use of a rapidly absorbable substitute, therefore, risks the robustness of the repair and puts patients at potential risk, as has been demonstrated in our case. The use of Ostene® in skull base repair applications is regarded by Baxter, the company producing Ostene® as off-label use; that is, it is not recommended or approved in Australia for this usage. Given the suggestion in the literature [5, 8–10] by way of direct comparison of the products, and general marketing, that Ostene® can be viewed as a substitute to traditional bone wax, we feel it is important to emphasise the pitfall of succumbing entirely to this thinking, particularly in a neurosurgical context. We remind ourselves that they are indeed different products with different applications. Real-world clinical problems with Ostene®, such as failing to maintain a sufficient air-tight seal, seem underreported. The lack of robust reporting on negative outcomes or complications associated with Ostene® may skew its perceived effectiveness in the literature. The neurosurgical community needs to engage in further discourse on the limitations of Ostene® in practice to provide a more balanced perspective.

Our case serves as a reminder of a limitation of soluble haemostatic agents, including bone wax substitutes, when dealing with situations that require a durable and long-term CSF seal. In these cases, conventional bone wax, despite its limitations, has a long history of high performance [2, 6, 7] and will remain a robust and appropriate option for the foreseeable future.

## Conclusion

For neurosurgical applications, the use of bioresorbable alternatives to traditional bone wax, such as Ostene® bone haemostasis material, needs to be considered carefully as they present unique pitfalls that may not be encountered in other surgical specialties. While these materials offer temporary haemostasis, they do not provide the durable and lasting barrier required in procedures such as air cell sealing during skull base repair. Despite its limitations, traditional bone wax remains a robust and reliable option in this context. This case underscores the need for increased awareness and further publication into the limitations of water-soluble bone haemostasis agents in neurosurgical practice.

## Data Availability

No datasets were generated or analysed during the current study.
